# How methodological frameworks are being developed: evidence from a scoping review

**DOI:** 10.1186/s12874-020-01061-4

**Published:** 2020-06-30

**Authors:** Nicola McMeekin, Olivia Wu, Evi Germeni, Andrew Briggs

**Affiliations:** Health Economics and Health Technology Assessment (HEHTA), Institute of Health and Wellbeing, University of Glasgow, Glasgow, G12 8RZ UK

**Keywords:** Methodological framework, Scoping review, Framework, Methodology

## Abstract

**Background:**

Although the benefits of using methodological frameworks are increasingly recognised, to date, there is no formal definition of what constitutes a ‘methodological framework’, nor is there any published guidance on how to develop one. For the purposes of this study we have defined a methodological framework as a structured guide to completing a process or procedure. This study’s aims are to: (a) map the existing landscape on the use of methodological frameworks; (b) identify approaches used for the development of methodological frameworks and terminology used; and (c) provide suggestions for developing future methodological frameworks. We took a broad view and did not limit our study to methodological frameworks in research and academia.

**Methods:**

A scoping review was conducted, drawing on Arksey and O’Malley’s methods and more recent guidance. We systematically searched two major electronic databases (MEDLINE and Web of Science), as well as grey literature sources and the reference lists and citations of all relevant papers. Study characteristics and approaches used for development of methodological frameworks were extracted from included studies. Descriptive analysis was conducted.

**Results:**

We included a total of 30 studies, representing a wide range of subject areas. The most commonly reported approach for developing a methodological framework was ‘Based on existing methods and guidelines’ (66.7%), followed by ‘Refined and validated’ (33.3%), ‘Experience and expertise’ (30.0%), ‘Literature review’ (26.7%), ‘Data synthesis and amalgamation’ (23.3%), ‘Data extraction’ (10.0%), ‘Iteratively developed’ (6.7%) and ‘Lab work results’ (3.3%). There was no consistent use of terminology; diverse terms for methodological framework were used across and, interchangeably, within studies.

**Conclusions:**

Although no formal guidance exists on how to develop a methodological framework, this scoping review found an overall consensus in approaches used, which can be broadly divided into three phases: (a) identifying data to inform the methodological framework; (b) developing the methodological framework; and (c) validating, testing and refining the methodological framework. Based on these phases, we provide suggestions to facilitate the development of future methodological frameworks.

## Background

There is no formal definition of a methodological framework amongst the academic community. There is, however, unspoken agreement that a methodological framework provides structured practical guidance or a tool to guide the user through a process, using stages or a step-by-step approach [1–5]. Specific descriptions of a methodological framework include: ‘a body of methods, rules and postulates employed by a particular procedure or set of procedures’ [6], a ‘set of structured principles’, an approach for ‘structuring how a given task is performed’ [7], and a ‘sequence of methods’.

The benefits of using methodological frameworks are manifold: they can improve the consistency, robustness and reporting of the activity [8], enhance the quality of the research, standardise approaches [5], and maximise trustworthiness of findings [2].

In 2017, Rivera et al. published the results of a literature review which identified existing methodological frameworks used to measure healthcare research impact and summarised the common themes and metrics used to measure this impact [6]. The authors found that the identified methodological frameworks had been developed using a variety of approaches, with no guidelines or consensus on the best pathway that should be used to develop a robust methodological framework. The authors concluded that this lack of guidance needs to be addressed to ensure that best practice methods can be used in the future*.* We sought to address this gap, by 1) systematically scoping the literature on methodological frameworks, charting and summarising approaches employed, and using these summarised approaches to make suggestions for developing future methodological frameworks, 2) identify terminology used in the literature in order to inform future research. Rather than limiting our search to methodological frameworks related to academic research as Rivera et al. did, we opted to be more inclusive so we could understand the rationale and approaches for the development of methodological frameworks in the wider arena.

## Methods

We carried out a scoping review as a way of mapping the existing landscape on the use of methodological frameworks, identifying approaches used to develop them, and summarising these approaches thematically to inform suggestions for developing methodological frameworks. Scoping reviews have been shown to be particularly useful for when a research area has not yet been widely reviewed, such as areas with emerging evidence [9], to examine the extent, range and nature of a research area [10], where there is a lack of consistency in methodology and terminology to clarify key concepts and definitions [11] and for informing a systematic review [12]. Our scoping review methodology followed Arksey and O’Malley’s recommendations [10], as well as more recent guidance by Levac [9] and Colquhoun et al., [11]. Our study consisted of the following stages: 1) identifying the research question; 2) identifying relevant studies; 3) study selection; 4) charting the data; and 5) collating, summarising and reporting the results. No publicly-available protocol is available for the research; however, interested readers can contact the corresponding author for further details on methods.

### Identifying the research question

There is no formal definition of a methodological framework, nor is there guidance on the approaches to use when developing a methodological framework. In this review the working definition of a methodological framework is a tool to guide the developer through a sequence of steps to complete a procedure. Methodology is defined as the group of methods used in a specified field, and framework is defined as a structure of rules or ideas. The primary research question posed in this review is ‘what approaches are used in developing a methodological framework and is there consistency in those approaches to enable making suggestions for developing methodological frameworks?’ The secondary research question is ‘what terminology is used for naming methodological frameworks?’

### Identifying relevant studies

Identifying relevant studies followed an iterative approach, guided by an experienced subject librarian. An initial search was conducted in August 2018 in Web of Science. The results of the initial search helped to inform the scoping review search. There were no standardised MESH terms for methodological frameworks, because of this index terms were also scrutinised.

The main scoping review search took place in September 2018. We searched MEDLINE and Web of Science for published literature and also conducted a search for grey literature. The search terms used were necessarily narrow to avoid an impractically large amount of potential studies. Only titles rather than abstracts were searched to ensure that the search terms were the main focus of the article or paper. Details of search terms used are included in Additional file 1.

The grey literature search used methods previously published by Godin’s et al. [13] who used systematic methods for grey literature searching. The search was conducted in Google and results were restricted to the first 10 pages (100 hits). A single search term was used; ‘Methodological framework development’. Drawing on the approach used by Rivera et al. [6], we also searched Google Images; methodological frameworks are often presented as a diagram and therefore could be easily identified using this approach. Based on Rivera et al’s published methods the first 50 items were screened [6]. The electronic search was supplemented by a manual search of the reference lists and citations of all the relevant studies.

### Study selection

Studies were eligible for inclusion if: (a) they included a methodological framework and reported the approach used for developing that framework; (b) were written in English; and (c) were published in the last decade (2008 onwards). Screening criteria were established a priori. Duplicates were removed, and titles and abstracts of identified papers were screened for potential eligibility by the first author (NM) after downloading the search results into Excel. The full texts of potentially eligible articles were retrieved and read to assess eligibility for final inclusion, also by the first author (NM). Any uncertainty over eligibility for inclusion was discussed by the authors.

### Charting the data

The lead author (NM) developed a data charting form on Microsoft Excel and extracted from each individual paper the following information: (a) basic study characteristics (i.e. authors, title, journal, type of study, year of study and country of origin); (b) subject area; (c) approaches taken in developing the methodological framework; and (d)terminology used for methodological frameworks .

### Collating, summarising and reporting the results

The extracted data were analysed in line with the aims of the scoping review. Approaches were examined in detail, then synthesised and grouped together into similar methods. The approaches are reported descriptively with frequencies and percentages. These approaches were then categorised into phases and interpreted to make the suggestions. The results were reported in line with the PRISMA Extension for Scoping Reviews (PRISMA-ScR): Checklist and Explanation [14]. The completed PRISMA-ScR is provided in Additional file 2.

## Results

### Literature search

**Fig. 1:**
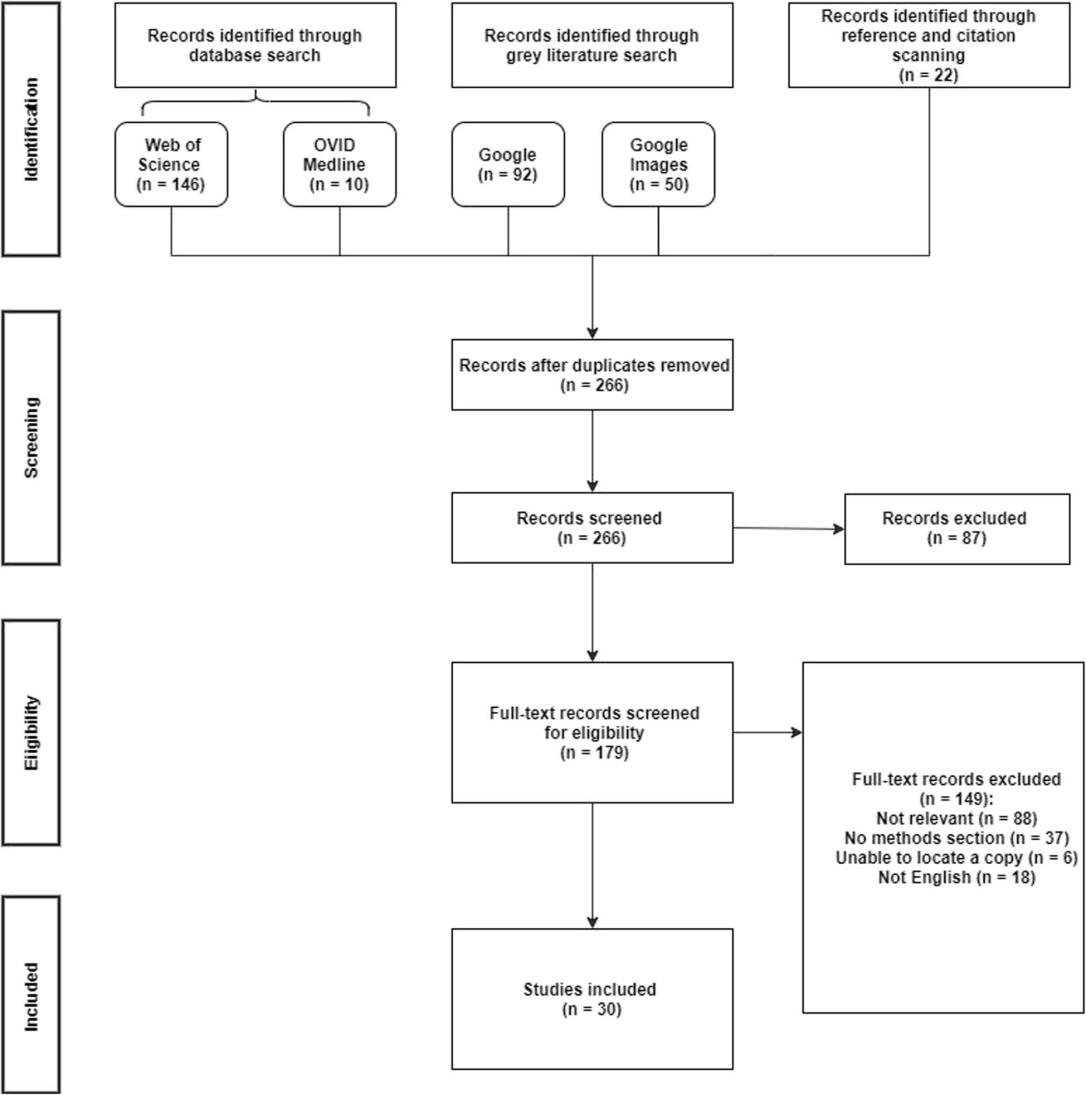
PRISMA flow chart of study selection

The combined search strategies yielded a total of 320 records (266 after removing duplicates). 179 potentially relevant full-text papers were screened and 30 were included in the review [1–5, 8, 15–38]. The flow chart of study selection is presented in Fig. 1.

### Study characteristics

A majority of included papers (26/30) were journal articles, followed by conference proceedings (3/30) and a book chapter (1/30). The studies represented a wide range of subject areas; 20 different subject areas were identified, the most common being ecology (6/30), followed by education (4/30), then manufacturing and regional (3/30), and healthcare, architecture and health economics (2/30). The papers originated from 14 countries; the most common was UK (8/30), followed by Greece, Germany, US and the Netherlands (3/30) and finally Italy (2/30). Basic study characteristics are presented in Additional File 3.

We found a variety of terms used to describe the methodological frameworks. This use of different terms was seen in both the title and the body of the study. Six studies did not include ‘methodological framework’ in the title (20.0%). Of these one included the words ‘methodological’ and ‘framework’ separately [2], four included only ‘framework’ in title and one used the term ‘conceptual framework’. Of these six studies two were identified from references [4, 5], two from citations [37, 38] and one from Google images [34].

**Fig. 2:**
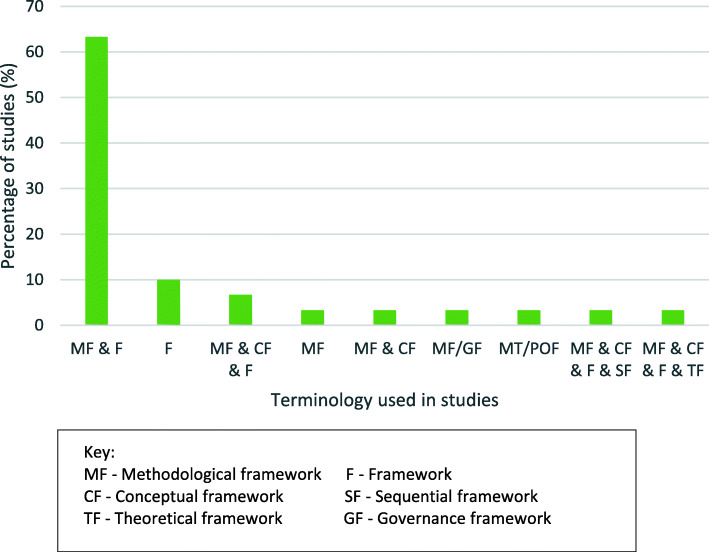
Terminology used in studies

Alternative terms for methodological frameworks were used interchangeably within the studies (Fig. 2).

Most studies included a combination of ‘methodological framework’ and ‘framework’ to describe the methodological framework (63.3%). One used a combination of methodological framework and conceptual framework. Three used ‘framework’ only and one used ‘methodological framework’ only. One study used three terms and a further two studies used a combination of four terms.

Keywords used in the studies that related to methodological frameworks are summarised in Table 1. Half of the studies (15/30) did not have any keywords related to methodological frameworks. Of those that used keywords related to methodological frameworks most used ‘methodology’ (4/30), followed by ‘methodological framework’ (3/30), ‘design methodology’ (2/30), ‘simulation methodology’ (1/30), ‘methods’ (1/30) and ‘guidance’ (1/30). One study contained two relevant keywords [5]. 4/30 studies had no keywords at all

**Table 1:** Keywords relevant to methodological frameworks extracted from studies

Keyword	Number (***n*** = 30)	Percentage %
None relevant to methodological frameworks	15	50.0%
Methodology	4	13.3%
N/A: no keywords in study	4	13.3%
Methodological framework	3	10.0%
Design methodology	2	6.6%
Simulation methodology	1	3.3%
Methods	1	3.3%
Guidance	1	3.3%

### Approaches used for the development of methodological frameworks

We identified eight different approaches used for developing methodological frameworks (Table 2), these are also summarised by study in Additional File 4.

**Table 2:** Approaches used for the development of methodological frameworks

Reported approaches	Number	Percentage (%)
Based on existing methods and guidelines	20	66.7
Refined and validated	10	33.3
Experience and expertise	9	30.0
Literature review	8	26.7
Data synthesis and amalgamation	7	23.3
Data extraction	3	10.0
Iteratively developed	2	6.7
Lab work results	1	3.3

The most frequently reported approach was ‘Based on existing methods and guidelines’, which comprise previous methodological frameworks or guidance and published methodology. Whilst some studies did not explain how the existing methods formed the foundations of the framework being developed, most did expand this further: adapting the methods [19, 24], integrating methods, building on the existing methods [4, 37], based on the framework [20–22, 27, 30, 33], combined well established guidelines which comprised the same stages [16], and the framework was basic inspiration [28]. Only one study specified how the frameworks or guidance was identified; Squires and colleagues used a literature review [5].

Ten studies reported ‘Refined and validated’ as a method. Approaches taken to refining and validating comprised; piloting the framework [35], trialling identified stages and using the results of the trial to further develop the framework [25], using a case study or Delphi panel to evaluate and refine the framework [5, 8, 33], using a case study to validate the framework [17, 29] and testing the framework [20]. Two studies did not report details of the case study [18, 24].

Nine studies reported using ‘Experience and expertise’ to develop the methodological framework, and reported using experience from different levels: personal [15], school/university [25] and country level [28]. One study restricted ‘experience’ to the authors’ experience [15], the rest included the experience of experts in the field of the methodological framework. In all but one study the experts were recruited specifically to develop the methodological framework, the remaining study used experience already reported [28]. Methods used to extract experience and expertise comprise: during meetings [18], consultations [39] and collaboration [33]. Two frameworks did not specifically mention experience but used surveys and interviews [34] and focus groups for extracting expertise [5]. Whilst these studies did not explicitly mention experience the methods reported would have extracted experience or views on experience.

Eight studies reported conducting a ‘Literature Review’. Specifically; purposeful sampling [2, 26], sources for searches included databases, dissertation [23], library catalogue, key author, databases websites and citations [8]. Other studies reported conducting a literature review but did not report specific methods used [5, 8, 23, 29, 33, 35].

Seven studies reported using ‘Data synthesis and amalgamation’. Specific methods included: identifying phases [2], themes [2, 34] and dimensions [23], analysing and grouping or categorising themes, or thematic analysis [2, 3, 8, 23, 26].

‘Data extraction’ was reported in three studies and includes extracting data from interviews and focus groups using transcribing methods [5, 34], and extracting key information from published literature [2].

‘Iteratively developed’ was a method reported in two studies, one framework had no details on this [20], the other explained that the framework evolved and developed as items were extracted, synthesised and revised [8].

The least frequently mentioned method was ‘Lab work results’, the study that reported using this method was from the field of explosives, where the results of lab tests were used to inform the framework [1].

A pattern emerged whilst reviewing the methods and in applying meaning to these results, they were split into three categories. The first category relates to identifying evidence or data to inform and shape the framework. This evidence comes from: existing methods, literature reviews, lab results and experience/expertise. The second category relates to developing the framework using the identified data, comprising: extracting data, and synthesising and amalgamating this data iteratively. The third and final category is refining and validating the framework: trialling the framework with pilot or case studies and or Delphi panels.

**Fig. 3:**
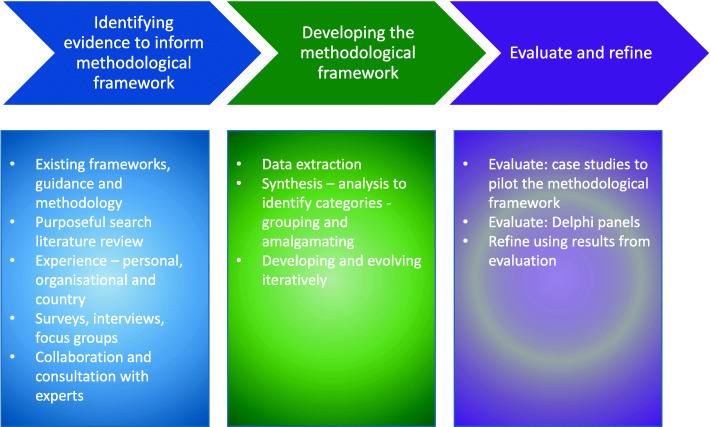
Summary of suggestions for developing methodological frameworks

The scoping review results were used as a basis for the following outline of suggestions that may be considered for developing a methodological framework on. The three phases underpinned the structure and specific approaches were included within those phases. These are summarised in Figure and explained in greater detail below.

(Uploaded as ‘Fig. 3 Summary of suggestions for developing methodological frameworks.pptx’)

### Phase 1 – identifying evidence to inform the methodological framework

This phase is split into two; the first is identifying previous frameworks or guidance which are used for the foundations of the new methodological framework, the second is identifying new data to help develop the methodological framework. This new data can be identified in numerous ways: purposeful literature searches, qualitative research (focus groups, interviews, surveys), collaboration between interested parties and the experience and expertise of the developers. If qualitative research is included, if possible it should be conducted with experts in the field of the methodological framework and not restricted to author experiences if possible.

### Phase 2 – developing the methodological framework

In this phase the frameworks or guidance identified in Phase 1 are adapted, combined with other guidance and built upon to create the foundations of the new methodological framework. Key information in the new data identified in Phase 1 should be extracted using appropriate methods. Appropriate methods include; transcribing qualitative data, entering themes into predesigned tables, and entering quantitative information into piloted data extraction forms. Once the information is extracted it should be analysed, synthesised, and grouped or amalgamated into categories to inform the new framework. This should be an iterative process; after grouping or amalgamation of the new data, it should be brought back to key experts and the study team for refinement. This iterative approach should be followed until consensus is reached on the proposed methodological framework.

### Phase 3 – evaluate and refine

In this final stage the proposed methodological framework should be evaluated and refined. Evaluation techniques include using case studies to pilot the methodological framework and Delphi panels. The results from this evaluation should be used to refine the methodological framework if appropriate. Refining will include updating the methodological framework with any changes identified from the evaluation stage and presenting these changes to key experts and the study team for verification.

These suggestions are not intended to be prescriptive, and the developer should adapt them to their specific situation. Finally, the developer should include the term ‘methodological framework’ at least in the title of the study, preferably in the body of the text too and as a keyword if possible.

## Discussion

### Summary of evidence

The purpose of this scoping review was to identify approaches taken in developing methodological frameworks and terminology used in describing them. We were able to locate 30 studies that were published in the last decade and reported these approaches. Studies covered 20 subject areas and came from 14 different countries. After synthesis and amalgamation, we identified eight approaches used for developing methodological frameworks. Not all studies with methodological frameworks reported the approaches used to develop them; out of 179 potentially eligible frameworks scrutinised in full, 37 (20.7%) were rejected because the authors did not report approaches, Studies which did report approaches were often not clear about the methods used. However, whilst the approaches used to develop methodological frameworks were not always reported or reported clearly, there were a sufficient number of common approaches to allow the amalgamation and categorisation of the approaches that were reported to form an evidence base on which suggestions for developing methodological frameworks could be made.

In the included studies extracted terms used to describe methodological frameworks highlighted the lack of clarity in terminology, as different terms were used to describe methodological frameworks within the studies. The majority of studies used a combination of ‘methodological framework’ and ‘framework’, which is understandable bearing in mind journal word limits and flow of discussions. Two studies used a combination of four terms highlighting the lack of clarity in terminology. This lack of clarity in terminology suggests that when conducting a literature search for methodological frameworks, it is likely that many methodological frameworks might not be identified. We recommend using ‘methodological framework’ in the title of the study as a minimum.

Many of the included studies did not use any keywords related to methodological frameworks suggesting that the studies were more focussed on the subject of the methodological framework rather than the actual process of developing the methodological framework itself.

As there is no existing guidance for developing methodological frameworks, it is not possible to interpret the results of this scoping review in light of what is already known. However, Rivera et al. [6] also concluded that methodological frameworks vary in their development, although there appear to be some common approaches. In their review, only one paper (4%) did not report any methods of development [40], compared to 37 (20.7%) in this review. Rivera et al. reported four key methods: using a literature review, stakeholders’ involvement, methods to incorporate stakeholder views and a pilot phase. The results from this scoping review identified additional methods, including: refined and validated, data synthesis, data synthesis and amalgamation and iteratively developed.

### Strengths and limitations

To the best of our knowledge, this is the first study to identify approaches used for the development of methodological frameworks; our work addresses an important gap in the literature by providing suggestions for the development of future methodological frameworks and highlighting issues with terminology which can inform future work. Further strengths are; the methodological frameworks identified and analysed come from many contexts and demonstrate a degree of natural variation, and our research offers a contemporary slice of how methodological frameworks are used.

Certain limitations need to be acknowledged and addressed. As with any review this research is limited by dependency on the quality of included studies and the search strategy, specific limitations are discussed further below [41].

First, issues with lack of consistency in terminology meant that further examples of methodological frameworks may have been missed in the search if a different term to ‘methodological framework’ had been used in the title. However, a pragmatic balance had to be struck between the sensitivity and specificity of the search; using the search term ‘framework’ only would have resulted in an impractical number of results. This limitation to the search strategy will have potentially resulted in limiting the number of approaches reported and limited the identification of variations in terminology Also, as previously discussed, not all the studies identified included methods, limiting the amount of data that could be extracted and included in the scoping review. Linked to this, not all methods were clearly reported, perhaps because of word count, the aim and focus of the paper, or traditionally how different disciplines report. Moreover, data screening and extraction was conducted by one reviewer, although key decisions on study selection were discussed with the wider team. Last, scoping reviews do not assess the quality of included evidence; therefore, there is a risk that the frameworks included in this review were not of high quality, however, as there is scant evidence in this area, a scoping review was the most suitable method to use [12, 42].

## Conclusions

The current lack of guidance provides an opportunity to make some initial steps towards addressing this gap in the knowledge. This scoping review summarises the reported approaches used in developing a methodological framework. This work can be viewed as the first step in developing robust guidance for developing a methodological framework. As the terminology, definitions and process are not widely agreed, there is a need for standardisation of these. Whilst terminology and definitions were not consistent, reported approaches for development were. This consistency allowed for suggestions to be made for developing methodological frameworks. Future research to update this scoping review and suggestions should include a systematic review based on the terminology identified, and collaboration with experts, for example using a Delphi panel or focus group, to develop best practise guidance. Furthermore, a standardised procedure to collecting qualitative data in phase one would add consistency and transparency to evidence gathering.

## Supplementary information

**Additional file 1.** OVID Medline search September 2018.

**Additional file 2.** Preferred Reporting Items for Systematic reviews and Meta-Analyses extension for Scoping Reviews (PRISMA-ScR) Checklist.

**Additional file 3.** Basic study characteristics.

**Additional file 4.** Extracted data from studies.

## Data Availability

Not applicable.
