# Supplementary material for: How methodological frameworks are being developed: evidence from a scoping review
Source: BMC Med Res Methodol. 2020 Jun 30;20:173. doi: 10.1186/s12874-020-01061-4 (PMC7325096; doi:10.1186/s12874-020-01061-4)
Supplement: Supplementary file 1 — Additional file 1. OVID Medline search September 2018. [file 12874_2020_1061_MOESM1_ESM.docx]

### Additional file 1

### OVID Medline search September 2018

|  | | |
| --- | --- | --- |
| 1. "develop*".m_titl. |  | |
| 2. methodological framework.m_titl. |  | |
| 3. "design*".m_titl. |  | |
| 4. "creat*".m_titl. |  | |
| 5. 1 or 3 or 4 |  | |
| 6. 2 and 5 Web of Science search September 2018 **#1 TITLE:** (methodological framework)  *DocType=All document types; Language=All languages*  **#2 TITLE:** (develop*) *OR* **TITLE:** (creat*) *OR* **TITLE:** (design*) *OR* **TITLE:** (writ*)  *DocType=All document types; Language=All languages;*  #3 #1 AND #2 |  | |
