# Supplementary material for: How methodological frameworks are being developed: evidence from a scoping review
Source: BMC Med Res Methodol. 2020 Jun 30;20:173. doi: 10.1186/s12874-020-01061-4 (PMC7325096; doi:10.1186/s12874-020-01061-4)
Supplement: Supplementary file 3 — Additional file 3. Basic study characteristics. [file 12874_2020_1061_MOESM3_ESM.pdf]

Additional file 3 - Basic Study Characteristics

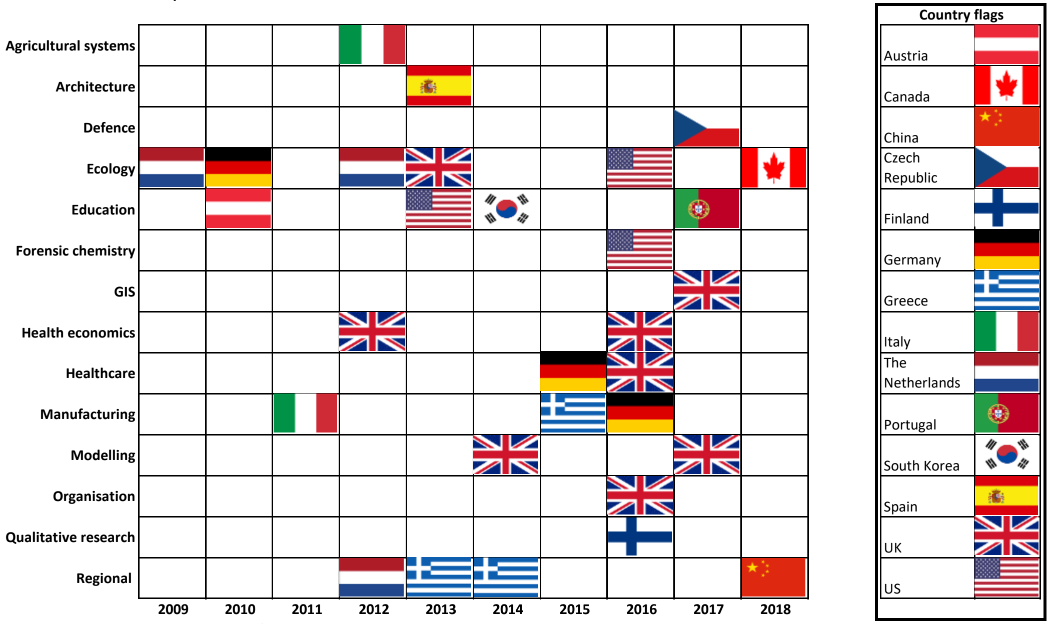

GIS: geographic information system

Source of flag images - all images were sourced from Wikimedia Commons		
Country	*Author*	*License*
Austria	Bundesministerium für Landesverteidigung	Public domain
Canada	Zscout370	Public domain
China	Skopp, redrawn by user Denelsom83 and user Zscout370	Public domain
Czech Republic	cs:-xfi-.	Public domain
Finland	Sebastian Koppehel	Public domain
Germany	Skopp and Madden	Public domain
Greece	CHANTEL	Creative Commons Attribution-Share Alike 3.0 Unported licence
Italy	ZScout370	Public domain
The Netherlands	Quistnix	Creative Commons attribution-Share Alike 3.0 unreported licence
Portugal	Marc Mongenet	Creative Commons Attribution-Share Alike 3.0 Unported license
South Korea	Reepy1	Public domain
Spain	CHV	Public domain
UK	Shanewithfreedom	Creative Commons Attribution-Share Alike 3.0 Unported license
US	Steinsplitter	Public domain
