# Supplementary material for: How methodological frameworks are being developed: evidence from a scoping review
Source: BMC Med Res Methodol. 2020 Jun 30;20:173. doi: 10.1186/s12874-020-01061-4 (PMC7325096; doi:10.1186/s12874-020-01061-4)
Supplement: Supplementary file 4 — Additional file 4. Extracted data from studies. [file 12874_2020_1061_MOESM4_ESM.docx]

**Additional file 4 – Extracted data from studies**

| **Characteristics** | | | **Reported approaches - methodology** | | | | | | | |
| --- | --- | --- | --- | --- | --- | --- | --- | --- | --- | --- |
|  | **Year** | **Country** | **Based on existing methods** | **Refined and validated** | **Experience and expertise** | **Lit review** | **Data synthesis and amalgamation** | **Data extraction** | **Iteratively developed** | **Lab work results** |
| Achillas(1) | 2014 | Greece |  |  | ✓ |  |  |  |  |  |
| Anagnostou(2) | 2016 | UK | ✓ |  |  |  |  |  |  |  |
| Battini(3) | 2011 | Italy |  | ✓ |  |  |  |  |  |  |
| Brondizio(4) | 2016 | US | ✓ |  |  |  |  |  |  |  |
| Chesson(5) | 2016 | US | ✓ |  |  |  |  |  |  | ✓ |
| Dean(6) | 2017 | UK |  | ✓ | ✓ |  |  |  |  |  |
| George(7) | 2012 | Italy | ✓ |  |  |  |  |  |  |  |
| Halbe(8) | 2018 | Canada | ✓ | ✓ |  |  |  |  | ✓ |  |
| Ianni(9) | 2013 | Spain | ✓ |  |  |  |  |  |  |  |
| Kallio(10) | 2016 | Finland |  |  |  | ✓ | ✓ | ✓ |  |  |
| Kumar(11) | 2018 | China | ✓ |  |  |  |  |  |  |  |
| Kumke(12) | 2016 | Germany | ✓ |  |  |  | ✓ |  |  |  |
| Lee(13) | 2014 | Korea |  |  |  | ✓ | ✓ |  |  |  |
| Linek(14) | 2010 | Austria | ✓ | ✓ |  |  |  |  |  |  |
| Lopes(15) | 2017 | Portugal |  | ✓ | ✓ |  |  |  |  |  |
| Nicod(16) | 2016 | UK |  |  |  | ✓ | ✓ |  |  |  |
| Pahl-Wostl(17) | 2010 | Germany | ✓ |  | ✓ |  |  |  |  |  |
| Panagiotopoulou(18) | 2014 | Greece | ✓ |  |  |  |  |  |  |  |
| Procházka(19) | 2017 | Czech Republic | ✓ |  | ✓ |  |  |  |  |  |
| Reed(20) | 2013 | UK | ✓ | ✓ |  | ✓ |  |  |  |  |
| Reidsma(21) | 2009 | Netherlands | ✓ |  |  |  |  |  |  |  |
| Rijke(22) | 2012 | Netherlands | ✓ |  |  |  |  |  |  |  |
| Rodgers (23) | 2016 | UK |  | ✓ |  | ✓ | ✓ |  | ✓ |  |
| Schmitt(24) | 2015 | Germany |  |  | ✓ |  |  |  |  |  |
| Stratigea(25) | 2013 | Greece | ✓ |  |  |  |  |  |  |  |
| Stremke(26) | 2012 | The Netherlands | ✓ | ✓ | ✓ | ✓ |  |  |  |  |
| Squires(27) | 2016 | UK | ✓ | ✓ | ✓ | ✓ | ✓ | ✓ |  |  |
| Sun(28) | 2013 | US | ✓ |  | ✓ |  | ✓ | ✓ |  |  |
| Tappenden(29) | 2012 | UK |  | ✓ |  | ✓ |  |  |  |  |
| Tondel(30) | 2014 | UK | ✓ |  |  |  |  |  |  |  |

1. Achillas C, Aidonis D, Iakovou E, Thymianidis M, Tzetzis D. A methodological framework for the inclusion of modern additive manufacturing into the production portfolio of a focused factory. Journal of Manufacturing Systems. 2015;37:328-39.

2. Anagnostou A, Taylor SJE. A distributed simulation methodological framework for OR/MS applications. Simulation Modelling Practice and Theory. 2017;70:101-19.

3. Battini D, Faccio M, Persona A, Sgarbossa F. New methodological framework to improve productivity and ergonomics in assembly system design. International Journal of Industrial Ergonomics. 2011;41(1):30-42.

4. Brondizio ES, Vogt ND, Mansur AV, Anthony EJ, Costa S, Hetrick S. A conceptual framework for analyzing deltas as coupled social-ecological systems: an example from the Amazon River Delta. Sustainability Science. 2016;11(4):591-609.

5. Chesson LA, Howa JD, Lott MJ, Ehleringer JR. Development of a methodological framework for applying isotope ratio mass spectrometry to explosive components. Forensic Chemistry. 2016;2:9-14.

6. Dean E, Taylor MJ, Francis H, Lisboa P, Appleton D, Jones M. A Methodological Framework for Geographic Information Systems Development. Systems Research and Behavioral Science. 2017;34(6):759-72.

7. George H, Bosc PM, Even MA, Belieres JF, Bessou C. WAW proposed methodological framework to monitor agricultural structural transformations and their contributions to sustainable development. Producing and reproducing farming systems New modes of organisation for sustainable food systems of tomorrow 10th European IFSA Symposium, Aarhus, Denmark, 1-4 July 2012. 2012.

8. Halbe J, Pahl-Wostl C, Adamowski J. A methodological framework to support the initiation, design and institutionalization of participatory modeling processes in water resources management. Journal of Hydrology. 2018;556:701-16.

9. Ianni M, de Leon MS. Applying Energy Performance-Based Design in Early Design Stages A methodological framework for integrating multiple BPS tools. Ecaade 2013: Computation and Performance, Vol 1. 2013:31-40.

10. Kallio H, Pietila A-M, Johnson M, Kangasniemi M. Systematic methodological review: developing a framework for a qualitative semi-structured interview guide. Journal of Advanced Nursing. 2016;72(12):2954-65.

11. Kumar A, Singh AR, Deng Y, He X, Kumar P, Bansal RC. A Novel Methodological Framework for the Design of Sustainable Rural Microgrid for Developing Nations. Ieee Access. 2018;6:24925-51.

12. Kumke M, Watschke H, Vietor T. A new methodological framework for design for additive manufacturing. Virtual and Physical Prototyping. 2016;11(1):3-19.

13. Lee J, Jang S. A methodological framework for instructional design model development: Critical dimensions and synthesized procedures. Etr&D-Educational Technology Research and Development. 2014;62(6):743-65.

14. Linek SB, Schwarz D, Bopp M, Albert D. When Playing Meets Learning: Methodological Framework for Designing Educational Games. Web Information Systems and Technologies. 2010;45:73-85.

15. Lopes AMB, Ruiz-Cecilia R. Designing Technology-Mediated Tasks for Language Teaching: A Methodological Framework. Hacettepe Universitesi Egitim Fakultesi Dergisi-Hacettepe University Journal of Education. 2017;32(2):265-79.

16. Nicod E, Kanavos P. Developing an evidence-based methodological framework to systematically compare HTA coverage decisions: A mixed methods study. Health Policy. 2016;120(1):35-45.

17. Pahl-Wostl C, Holtz G, Kastens B, Knieper C. Analyzing complex water governance regimes: the Management and Transition Framework. Environmental Science & Policy. 2010;13(7):571-81.

18. Panagiotopoulou M, Stratigea A. A participatory methodological framework for paving alternative local tourist development paths-the case of Sterea Ellada Region. European Journal of Futures Research. 2014;2(1).

19. Procházka J, Melichar J. Methodological Framework for Operational Risk Assessment. Vojenské rozhledy. 2017;26:19-34.

20. Reed MS, Kenter J, Bonn A, Broad K, Burt TP, Fazey IR, et al. Participatory scenario development for environmental management: A methodological framework illustrated with experience from the UK uplands. Journal of Environmental Management. 2013;128:345-62.

21. Reidsma P, Konig H, Feng S, Bezlepkina I, Keulen Hv, Ittersum MKv, et al. A methodological framework for sustainability impact assessment of land use policies in developing countries: re-using and complementing approaches. Proceedings of the Conference on integrated assessment of agriculture and sustainable development: Setting the Agenda for Science and Policy (AgSAP 2009), Hotel Zuiderduin, Egmond aan Zee, The Netherlands, 10-12 March 2009. 2009:138-9.

22. Rijke J, Brown R, Zevenbergen C, Ashley R, Farrelly M, Morison P, et al. Fit-for-purpose governance: A framework to make adaptive governance operational. Environmental Science & Policy. 2012;22:73-84.

23. Rodgers M, Thomas S, Harden M, Parker G, Street A, Eastwood A. Developing a methodological  framework for organisational case studies: a rapid review and consensus development process. Health Serv Deliv Res. 2016;4(1).

24. Schmitt J, Apfelbacher C, Spuls PI, Thomas KS, Simpson EL, Furue M, et al. The Harmonizing Outcome Measures for Eczema (HOME) Roadmap: A Methodological Framework to Develop Core Sets of Outcome Measurements in Dermatology. Journal of Investigative Dermatology. 2015;135(1):24-30.

25. Stratigea A, Papadopoulou CA. Foresight Analysis at the Regional Level - A Participatory  Methodological Framework  Journal of Management and Strategy. 2013;4(2).

26. Stremke S, Van Kann F, Koh J. Integrated Visions (Part I): Methodological Framework for Long-term Regional Design. European Planning Studies. 2012;20(2):305-19.

27. Squires H, Chilcott J, Akehurst R, Burr J, Kelly MP. A Framework for Developing the Structure of Public Health Economic Models. Value in Health. 2016;19(5):588-601.

28. Sun Y, Strobel J. Elementary Engineering Education (EEE)  Adoption and Expertise Development Framework:  An Inductive and Deductive Study. Journal of Pre-College Engineering Education Research (J-PEER). 2013;3(1).

29. Bafutto M, Costa MBG, Silva KTPE, Costa JPV, Reviglio CCSO, Oliveira EC, et al. Vitamin D is related to the effects of anti-TNF treatment in Crohn's disease patients. United European Gastroenterology Journal. 2017;5 (5 Supplement 1):A529.

30. Tondel K, Niederer SA, Land S, Smith NP. Insight into model mechanisms through automatic parameter fitting: a new methodological framework for model development. Bmc Systems Biology. 2014;8.
